# Insight into the Tropism of Dengue Virus in Humans

**DOI:** 10.3390/v11121136

**Published:** 2019-12-09

**Authors:** Feroza Begum, Sandeepan Das, Debica Mukherjee, Sweety Mal, Upasana Ray

**Affiliations:** 1 CSIR-Indian Institute of Chemical Biology, 4 Raja S.C. Mullick Road, Jadavpur, Kolkata700032, India; bferoza24@gmail.com (F.B.); sandeepandaszoo@gmail.com (S.D.); debicamukherjee3@gmail.com (D.M.); sweetymal1994@gmail.com (S.M.); 2 Academy of Scientific and Innovative Research (AcSIR), Ghaziabad 201002, India

**Keywords:** DENV, tropism, receptors

## Abstract

In tropical and subtropical zones, arboviruses are among the major threats to human life, affecting a large number of populations with serious diseases. Worldwide, over three hundred million people are infected with dengue virus (DENV) every year as per the World Health Organization (WHO). DENV-mediated disease severity ranges from a mild fever to hemorrhagic fever and shock syndrome. Patients suffering from severe infection might experience multi-organ failure, cardiomyopathy and even encephalopathy, further complicating the disease pathogenesis. In life-threatening cases, DENV has been reported to affect almost all organs of the human body. In this review, we discuss the organ tropism of DENV in humans in depth as detected in various autopsy studies. Keeping in mind the fact that there is currently no DENV-specific antiviral, it is of utmost importance to achieve a vivid picture of the susceptible cells in humans which might help in designing antivirals against DENV, especially targeting those tissues in which infection might lead to life-threatening conditions.

## 1. Importance

Currently, there is no DENV-specific antiviral treatment available in clinics. Virus entry is the first step of the viral life cycle and marks the beginning of the pathogenic mechanism. Every complicated molecular activity that might occur inside the host cells primarily depends on the success of the viral particle in entering its target cell. A vivid picture of the sensitive cells might help in screening the primary tissue/cell types that the virus infects more often or shows a higher rate of pathogenesis in. This would encourage a detailed receptor analysis so as to gather information with respect to all possible receptors/co-receptors that the virus is capable of using in order to gain cellular entry. In depth knowledge about the target cells and the receptor usage would help designing antivirals against DENV, targeting tropism and preventing the progression of the fever to severe infection or preventing extensive infection of the vital organs by this virus.

## 2. Introduction

Dengue virus (DENV) belongs to the virus family Flaviviridae under the genera *Flavivirus*, which include arboviruses such as Zika virus (ZKV), Japanese encephalitis virus (JEV), West Nile virus (WNV), Yellow Fever Virus (YFV) and others. DENV is an enveloped virus, with a virion size of 50nm, and contains a positive-sense single-stranded RNA of approximately 11kb in size, packaged by viral capsid (C) proteins. The genome has one open reading frame (ORF) and encodes a single polyprotein that is further processed to generate three structural proteins, namely capsid(C), pre-membrane/membrane (prM/M) and envelope (E), and seven non-structural proteins, namely NS1, NS2A, NS2B, NS3, NS4A, NS4B and NS5 [1]. The envelope of the virus is a host-derived lipid bilayer, surrounded by 180 copies of two different glycoproteins (prM and E) [2]. DENV is classified into four different serotypes (DENV 1–4) based on antigenic heterogeneity and its serotypes can be further distinguished into different genotypes [3].

In urban areas, the natural host of DENV is human and is transmitted by two arthropod vectors of the genus *Aedes*, *Aedes aegypti* and *Aedes albopictus.* DENV infection is endemic in hot climatic regions of the world and according to WHO 2017 reports, an estimated 390 million DENV infection cases per year have been reported—of which, 96 million cases display the symptoms of the disease [4]. The acuteness of the infection depends on various factors such as virus strain and its virulence, age of the individual, sex, high BMI, immune status and host genetics [1,5,6,7]. It leads to asymptomatic infection in the majority of cases but, in some circumstances, it may lead to self-healing, mild flu-like symptoms known as Dengue fever (DF) or other severe forms of the disease such as Dengue hemorrhagic fever (DHF). DHF is characterized by coagulopathy, increased vascular fragility, plasma leakage into interstitial spaces, thrombocytopenia, and hemorrhage, which may progress to a hypovolemic shock called dengue shock syndrome (DSS), categorized as a grade 3 or grade 4 DHF according to the WHO [3,8,9].

In this review article, we shed light on human autopsy studies performed on DENV-infected patients and the various cells capable of being infected. We also aim to discuss the cells in different organs where DENV might replicate and further disseminate, as investigated in various in vitro and in vivo studies. The entry point of DENV in humans is an underexplored field which needs to be studied in greater details. Knowledge about the tropism of DENV can potentially help in designing DENV-specific antivirals or therapeutics.

## 3. Dengue Tropism in Humans

Researchers have observed a marked difference in DENV replication between different cell lines and cells in the infected host. DENV replicates in many cell lines but in vivo, DENV replicates only in few cell types [5,10,11,12]. Noisakran et al. explained this strikingly different observation considering that since IFN signaling plays a very important role in controlling DENV permissiveness in the infected host, immortalized cell lines used to study DENV infections are deficient in IFN response and hence are highly permissive to DENV. Therefore, it is important to study DENV-infected tissue samples to achieve a proper insight to the cells that are targeted by the virus naturally.

Different laboratories worked to decipher the exact replicative sites of DENV in humans by studying the tissue samples of infected humans and mice models. Scientists found different results—a few cells were found to support DENV replication consistently, while others were found to show varying susceptibility to DENV infection. However, the most extensively studied organs/tissues where DENV was not only present but also replicated are skin, peripheral blood, spleen, lymph node and liver [5,13,14,15,16,17,18,19,20]. DENV-infected human autopsy tissues were studied to detect the presence of various DENV antigens in different organs and also to check whether DENV also replicates in these tissues. Presence of DENV (−) sense RNA or NS3/NS5 proteins in a particular cell may indicate DENV replication, as these antigens appear when DENV undergoes replication. On the other hand, the detection of other DENV antigens (E, prM, C, (+) sense DENV RNA) does not indicate the active replication of DENV in the cells, as cells may non-specifically take up viral RNA and other antigens from the surroundings but may not allow DENV to replicate. In this article, we aim to discuss the various organs and tissues in humans that have been studied to possess DENV antigens as seen in autopsy studies. We start exploring DENV tropism in different organs based on their importance in pathogenesis and viral spread.

## 4. Skin

Being the first barrier to pathogens, skin has a primary role to play in an innate immune response. The cellular diversities in skin provide a suitable environment for DENV to replicate and disperse in the entire body [10,21,22,23,24]. It has been suggested by different authors that DENV is directly injected in the dermis layer of skin instead of the epidermis, as, during imbibition, 50% of the mosquito fascicle penetrates into the skin [10].

Briefly after the mosquito bite, DENV first enters the skin and infects both the dermal and epidermal resident cells [21,25,26,27,28,29,30,31]. The skin cells which are known to play a role in DENV tropism include Langerhans cells (LC), dermal macrophages, blood-derived monocytes, dermal DCs (CD1c+ and CD14+), keratinocytes, endothelium, fibroblast and mast cells [21,25,26,27,28,29,30,31]. Wu et al. and Ho et al. were among the first groups to show that LCs and dermal DCs, along with the blood-derived DCs are infected by DENV and support its replication [25,26]. Cerny et al. and Schaeffer et al. studied healthy human skin and found dermal DCs (CD1c+ and CD14+), LCs and dermal macrophages to be infected by DENV [21,27]. Further studies performed on the dynamics of DENV infection in human skin explants identified LCs, macrophages, dermal DCs, mast cells, fibroblasts, keratinocytes and lymphatic endothelium to be infected and detected DENV NS3 protein in the infected cells [28].

Isolated mature mast cells from a healthy human skin were also found to be infected by DENV [31]. Furthermore, the infected mast cells have been observed to generate infectious extracellular granules which carry DENV via lymph to the various lymphoid organs, leading to its further spread [31]. Hence, these cells in the skin do not only express DENV antigen but also allow DENV to replicate and dissipate in the host.

Studies on the route of DENV spread in the host following mosquito imbibition states the important role of CD1c+ DCs, CD14+ DCs, dermal macrophages, LCs and mast cells in the process [31,32,33]. After these cells (CD1c+ DCs, CD14+ DCs, dermal macrophages, LCs) are infected, they leave the site to migrate to the lymphoid organs and to maintain a fresh pool of these cells in the skin, and the precursor cells (blood-derived monocytes and blood-borne LC precursors) differentiate, hence providing a fresh host for DENV [32,33]. The lymph nodes and spleen are infected by these migrating cells and the organ resident cells further undergo infection. DENV replicates in these cells and spreads in the lymphatic system, ultimately infecting the blood-derived monocytes and myeloid DCs, which further disseminate the virus to the non-lymphoid organs [3,19].

## 5. Lymph Node and Thymus

Lymph node is another lymphoid organ exploited by DENV, where extensive DENV infection has been reported [19]. Various cells in the lymph nodes of infected humans that were found to be infected by DENV and probably support DENV replication are macrophages, immunoblasts, lymphocytes, plasma cells, reactive germinal centers and mononuclear cells [13,18,34,35].

Viral RNA was found to be localized in the macrophages of lymph nodes and skin of a DHF patient in Thailand [13]. Immunoblasts, lymphocytes, plasma cells and macrophages in DENV-infected human lymph nodes were also found to be DENV positive in immunoperoxidase staining with anti-DENV antibodies [34]. NS3 immunostaining by Balsitis et al. revealed macrophages in the reactive germinal centers of the lymph node as infected by DENV in humans [18]. Further studies detected the presence of DENV antigens (NS1 and E) in the germinal centers of lymph node and antigens such as NS1, NS3 and E were found in the mononuclear cells of follicular and inter follicular regions [35]. Hence, in lymph node mononuclear cells in the germinal centers, follicular and inter follicular regions appear to be the replicative site of DENV. However, Jessie et al. did not find DENV in the lymph nodes of DENV-infected human samples [15].

Unlike other lymphoid organs, the role of thymus in DENV tropism has not been investigated much and very few human autopsy tissue studies observed thymus to be infected by DENV. Thymus tissue from the autopsy of a DENV-infected patient showed positive DENV staining in certain regions [36]. However, in later autopsy studies, Jessie et al. and others failed to detect DENV antigens in thymus [15]. Hence, it is surprising to find a less important role of thymus in DENV infection as other lymphoid organs, i.e., spleen, lymph node, bone marrow and Peyer’s patches are known to possess DENV in an infected host.

## 6. Spleen

Spleen is among the major secondary lymphoid organs which perform important functions in our body, particularly blood purification, the capture of foreign antigens from blood, hematopoiesis and storage of platelets. The mononuclear cell composition in the spleen was found to be significantly different from that of PBMC [37]. Studies on tissues obtained from the necropsy of DENV-infected patients have shown DENV to infect and replicate in different cells of the spleen. Mononuclear cells (DC and macrophages) in the red and white pulp regions, germinal centers of the lymphoid follicle, lymphoid cells in the red pulp, binucleated and multinucleated giant cells of the splenic red pulp, immunoblast and centroblast-like cells in the white pulp region, sinusoidal and splenic endothelium were found to be infected by DENV and supported DENV replication as detected by the presence of nonstructural proteins (NS1,NS3), DENV E protein and DENV RNA [15,18,20,34,35,37].

Earlier studies on the spleen of infected humans observed DENV antigens in cells such as immunoblasts, lymphocytes in the white pulp, plasma cells and macrophages in red pulp [34]. Based on immunohistochemistry (IHC) and in situ hybridization (ISH) techniques, viral antigens and viral RNA were also observed in macrophages, lymphoid cells, binucleated and multinucleated giant cells of splenic red pulp, germinal centers of lymphoid follicle, immunoblast and centroblast-like cells in the white pulp region of the spleen [15]. Primary human splenic macrophages (CD14+CD3- CD19-) were found to be the target of DENV in vitro both in the presence and absence of anti-DENV antibodies as DENV antigen (E) and viral RNA were detected in these cells 2days p.i. [37]. Balsitis et al. observed NS3 antigens in mononuclear phagocytic cells (macrophages and DCs) of red pulp and white pulp, splenic endothelium and sinusoidal endothelium of autopsy tissues [18]. Further studies on autopsy samples identified DENV antigens (NS1, NS3 and E) in the mononuclear cells of red and white pulp regions in spleen [35]. Hence in the spleen, monocytes/macrophages, DCs, splenic and sinusoidal endothelium may be considered to be the true targets of DENV which support DENV replication.

## 7. Liver

Some studies documented the involvement of liver in DENV infection because of a clinical manifestation known as hepatomegaly, i.e., the abnormal enlargement of the liver, with some reports suggesting a frequency of 50–100% of hepatomegaly, and others suggesting a relatively lower rate of hepatomegaly in DENV infection [38,39,40,41,42,43,44,45,46,47,48]. Also, The serum glutamic pyruvic transaminase (SGPT) and serum glutamic oxaloacetic transaminase (SGOT) levels were found to be higher in DHF patients than DF patients [49].

In several studies, DENV particles were recovered from different cells of the liver, from both pre- and post-mortem liver specimens [15,18,50,51,52,53,54,55]. Autopsy tissues of DENV-infected human liver revealed liver to be highly infected and supportive to DENV replication. Mostly hepatocytes, Kupffer cells and endothelium were found to be positive for (−) and (+) sense DENV RNA and NS3 proteins [13,20,34,56]. Jessie et al. could detect Dengue antigen in hepatocytes, Kupffer cells, endothelium, lymphocytes, and monocytes of the vascular lumen in liver [15]. However, other researchers detected DENV antigens mainly in the hepatocytes and to a lesser extent in other cells of the liver [18,50,51,52,53,54,55]. These contradicting observations might arise due to the difference in tissue tropism of DENV lineages or due to the difference in protocols followed and methodologies of sample preparations. Kupffer cells have been shown to be DENV positive by other researchers using anti-DENV E antibodies [15,52,54]. However, Balsitis et al. were unable to detect DENV NS3 protein in Kupffer cells, suggesting the possible non-specific uptake of DENV antigens or simple adsorption of the virus antigens on the cells [18]. Work by Marianneau and colleagues further showed that DENV is efficiently taken up by the Kupffer cells without the production of viral progeny [57]. Hence further studies are required to highlight the roles of Kupffer cells and endothelial cells in DENV tropism and pathogenesis.

## 8. Bone Marrow

Bone marrow suppression is among the pathogenic manifestations of DENV infection, which includes reduced megakaryocytopoiesis, granulopoiesis and erythropoiesis in the early days of infection (before the 4th day of fever), but after a few days of infection (after the 4th day of fever) hyperplasia was observed in megakaryocytes, myeloid cells and erythroid cells [58,59]. In a few reports, DENV2 and viral RNA were isolated from DENV-infected necropsy bone marrow samples [60,61]. Also, in a study conducted by Jessie at al., a small number of cells in the bone marrow were found to be DENV permissive and DENV antigens were detected only in myeloid cells but not in megakaryocytes [15]. It was demonstrated that in the long-term bone marrow culture, DENV2 can infect and replicate in the stromal cells [59]. Later studies depicted two distinct populations of DENV-positive stromal cells; one which was relatively small and CD11b/CD18 positive, while the second type was large, expressing nerve growth factor receptor and identified as adventitial reticular cells [62,63]. Further, it was shown that DENV could propagate in human bone marrow cells and hematopoietic cell lines, and the cells showing erythroid characters were more DENV permissive [64]. In the same paper, researchers had shown a serotype-specific infection in the bone marrow, where DENV4 could propagate more readily in erythroid cells than in non-erythroid cells, but DENV2 was primarily found in non-erythroid cells and to a lesser extent in erythroid cells [64]. Another case of DENV infection in a bone marrow transplant patient has been reported, where the recipient died due to secondary DENV4 infection acquired from the donor during bone marrow transplantation [65]. Hence, the current knowledge regarding DENV infection of bone marrow is scant, making it difficult to understand its exact role in the infectious process.

## 9. Central Nervous System (CNS)

Neurological complications in DENV are rare, accounting for 4–5% of the confirmed DENV-infected cases [66]. Furthermore, neurological complications associated with DENV infection can be of different types such as encephalopathy, encephalitis, muscle dysfunction, neuro-ophthalmic disorder and immune-mediated syndromes [67] and CNS has been reported to be involved in DENV infection, as seen in various autopsy studies. Furthermore, DENV RNA has been isolated from the CSF of DENV-infected patients manifesting neurological complications, irrespective of the stage (primary or secondary) and severity of the disease (DF, DHF, DSS).

Most of the DENV-infected patients showing neurological abnormalities were positive for DENV antigens (RNA, NS1) in the CSF as detected via RT-PCR, and ELISA [66,68,69,70,71,72,73]. DENV-specific antibodies (IgG and IgM) were also detected in the CSF to confirm DENV infection in the CNS [66,68,69,70,71,72,73].

Immunoperoxidase studies on DENV-infected necropsy samples revealed the presence of DENV in neurons of the cerebrum, a few lymphocytes in vessels, Purkinje cells and some granular cells in the cerebellum, astrocytes, microglia, epithelium lining choroid plexus and vessels [34]. However, cells supporting DENV multiplication were not studied further by this group. Miagostovich et al. showed the presence of DENV antigen in the CNS (brain) of DENV-infected patients and found CD68+ macrophages to localize in the brain sections [14]. They predicted the role of DENV-infected macrophages in carrying DENV to the brain and causing DENV-mediated encephalitis. Hence, this might be among the ways that DENV crosses the blood-brain barrier and traverses to the brain to establish the infection further. Autopsy studies by Ramos et al. showed similar results and presence of DENV was observed in neurons, astrocytes, microglia and endothelial cells (ECs) in medulla and cerebellum as assessed by RT-PCR and immunostaining using anti-DENV antibodies [74]. DENV antigen was also detected in the autopsy brain tissue of the patient by RT-PCR and DENV-specific antibodies [75]. Other immunohistochemical analyses of DENV-infected human brain using anti NS3 antibodies revealed the presence of NS3 in the perivascular astrocytes and peripheral blood monocytes/macrophages (PBMCs) in cerebrum but not in other cells of the brain as reported previously (such as ECs, neurons and glial cells) [18].

## 10. Kidney

Various symptoms indicating the possible involvement of kidney in DENV infection have been reported which includes proteinuria, hematuria, various types of glomerulonephritis, acute kidney injury (AKI), elevated serum creatinine level, acute tubular necrosis and hemolytic uremic syndrome [15,20,76,77,78,79,80,81,82,83,84,85,86,87,88,89,90,91,92,93,94,95]. Nevertheless, the importance of this organ in sustaining DENV infection has not been well explored. Fewer autopsy studies have explicated the understanding of the role of kidney in DENV infection [15,20,34]. Bhoopat et al. could detect DENV antigens in various cells of DENV-infected human kidney tissue such as immunoblasts, histiocytes, plasma cells and lymphocytes by using immunoperoxidase technique and monoclonal antibodies against DENV [34]. Viral antigens were also found in the kidney tubular cells but RNA was not detected, which suggests that the presence of viral antigens might be due to the engulfment of the immune complex [15]. Likewise, DENV antigens were also observed in the kidney of a DENV-infected patient, without any sign of viral RNA replication [20]. Hence, the role of kidney cells in supporting DENV infection needs to be further studied in order to better understand their importance in DENV dissemination.

## 11. Lungs

As with other organs, DENV particles, viral RNA and virus-like particles were also detected in the necropsy lung sample of DENV fatal cases [60,96,97]. DENV infection is known to cause lung abnormalities such as pulmonary embolism, pulmonary edema, interstitial edema, diffuse alveolar congestion and hemorrhage [98]. However, the replication of DENV in this organ has not been well studied. Few reports state the importance of certain cells in the lungs in supporting DENV replication [14,15,18,20,34,50]. DENV antigens were detected using anti-DENV antibodies in lung tissues of infected patients and the presence of negative strands of DENV RNA and NS3 indicates that lung could be another organ supporting DENV replication. Specifically, cells such as alveolar macrophages, type II pneumocytes and endothelium were found to be infected [14,20,34]. DENV antigen NS3 was also detected in alveolar macrophages in autopsy lung tissue sample [15,18,50].

## 12. Heart

Common cardiac manifestations during DENV infection include rhythmic abnormalities—mostly, sinus bradycardia, pericarditis and myocarditis [99,100,101]. Some rare cases of complete heart block during DENV infection has also been reported [102,103]. Another case of the manifestation of acute myocarditis leading to cardiogenic shock and death during DENV infection has been reported and an electron microscopy study in a necroscopic sample revealed the presence of clusters of viral particles in cardiomyocytes and interstitial space [100]. As with other DENV-infected organs, DENV was also detected and isolated from the autopsy sample of heart [60,96]. In most of the cases, DENV2 antigens were found in the mononuclear cells of the heart [20,50,100]. DENV antigens (NS3 and negative sense viral RNA) were also detected in myocardial fibers, endothelial cells and myocardial interstitial cells, confirming viral replication in these cells [20,100,104].

## 13. Tropism in Mouse

The strong preference of DENV for human hosts has been linked to its ability to perturb IFN response in humans but not in other animal models such as mice and non-human primates. The non-structural proteins of DENV such as NS2a, NS2b, NS4a, NS4b and NS5 have been studied to interfere with the type I interferon signaling pathway in humans by either interfering with the IFN signaling pathway or blocking IFN production, thereby leading to a productive infection in their natural host. Particularly, the NS5 protein of DENV degrades STAT2 protein in humans but fails to act on mouse STAT2 and is hence unable to infect mice [105,106]. Also, it has been shown that the STING protein, which is the stimulator for the interferon gene, is cleaved by the DENV NS2b3 protease in humans but not in mouse and other primates [107,108,109].

As the IFN response in mice helps in the clearance of DENV, researchers have tried to develop an immune-compromised mice model deficient in IFN-α /β and -γ receptors (AG129) or deficient in IFN-α /β receptor only (A129) in the 129/Sv background to study DENV pathogenesis [16,18,19,110,111,112]. They are highly susceptible to DENV infection, resulting in high levels of viremia and vascular leakage [113,114]. Recent studies have also shown organ damage, vascular damage and cytokine storm in AG129 mice when inoculated with a non-mouse-adapted DENV2 strain via the i.p. route [115,116]. AG129 mice model along with the humanized mouse model (immune-deficient mice transplanted with human umbilical cord stem cells, NOD-scid IL2Rc^null^ mice) are currently used to study DENV tropism and antiviral response [16,117,118].

Due to the difficulties in obtaining human autopsy tissues, researchers have tried to develop DENV susceptible mice models and study them to better understand the tropism and pathogenesis of DENV infection. Tropism studies in mice models correlate with human autopsy studies and have helped in exploring and enlarging our knowledge in this field.

DENV tropism-based studies in mice models revealed varying results as in human studies. DENV antigens were detected in the skin, liver, spleen, lymph nodes, kidney, bone marrow, lung, thymus, brain, stomach, and intestine of the infected mice models as studied by various researchers and the results were similar to DENV-infected human autopsy studies [16,18,19,90,111,112,119]. The same primary targets were identified by other authors when they studied DENV infection in the humanized mouse model and concluded the presence and replication of DENV2 in blood along with its presence in monocytes, macrophages, T and B cells in spleen and in bone marrow [118]. Furthermore, studies by another group highlighted the essential role of hematopoietic cells in DENV pathogenesis and indicated that the hematopoietic cell lineages are the most predominant cells used by DENV2 to replicate in the host [120].

**Figure 1:**
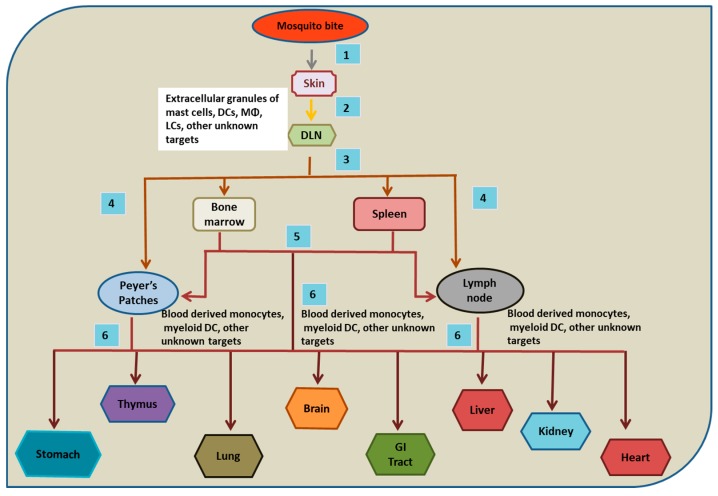
Probable Route of DENV dissemination in humans. (**1**) and (**2**) After mosquito bites, cells of the skin are infected and carry DENV to the draining lymph nodes (DLNs) via lymph. (**3**) From DLNs, resident macrophages (and other unknown cells) are infected and carry the virus to the lymphatic and vascular system, leading to the infection of bone marrow and spleen. (**4**) and (**5**) Later, Peyer’s patches and lymph nodes are infected and might receive DENV directly from DLNs or via bone marrow and spleen. (**6**) At later time points of infection, various non lymphoid organs are likely infected. However, the exact mechanism and mode of the dissemination of DENV to various organs are not precisely understood.

Researchers have used the AG129 mouse model to decipher the sequence in which specific tissues and organs are targeted in DENV infection by using a fluorescent immunohistochemistry (FIHC) technique. It was observed in vivo that lymphoid organs are the primary sites targeted by DENV for its replication followed by the nonlymphoid organs. Within a period of 12–48 h after intravenous DENV infection in mice, the extensive replication of DENV was detected in spleen, bone marrow, lymph nodes, and Peyer’s patches, followed by a high level of DENV NS3 expression in thymus and non-lymphoid tissues within 72 h of DENV infection (particularly in liver, heart, kidneys and GI tract) [19]. The authors further infected AG129 mice via a peripheral route (intra foot pad inoculation) and discovered draining lymph nodes (DLNs) to be the first target where DENV replicated and later entered the blood circulation [19]. Through blood, it was found to gain access to other lymphoid and non-lymphoid tissues [19]. Hence, the findings on the infection dynamics in mice can be considered together with the tropism study in humans to generate a proposed route of DENV dissemination in humans (Figure 1).

## 14. Discussion

**Figure 2:**
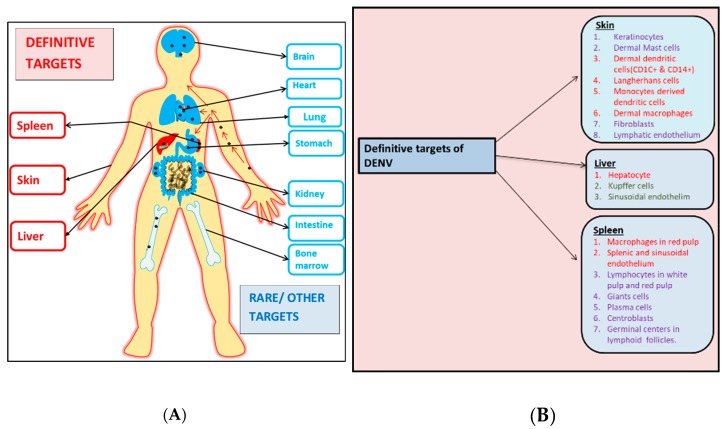
A diagrammatic representation of the various organs and their respective cells that are infected by dengue virus (DENV). (**A**) This panel represents the definitive (written in red) and the probable/rare targets (written in blue) of the virus during the infection process. (**B**) Specific cellular subsets of the definitive organs infected by DENV. Cells marked red have been consistently shown to be infected by DENV where the virus also replicates. Cells marked green have also been found to be targeted by DENV albeit inconsistently. Cells marked purple were found to be DENV positive in few autopsy studies but did not show any signs of DENV replication.

Studies on the autopsy tissue samples of DENV-infected patients have increased our knowledge regarding DENV tropism and pathogenesis in its natural host. However, research has so far failed to highlight the specific cells in the organs that were absolutely necessary for DENV replication. Important organs and tissues in humans where DENV is found to extensively multiply are blood, skin, spleen, lymph node and liver as shown in Figure 2. Other important organs (brain, thymus, heart, kidney, GI tract and bone marrow) showed contradictory results in autopsy studies and hence further inspection is needed to determine their role in DENV dissemination (Table 1). From the above-mentioned autopsy studies, different cells (Figure 2) have been found to consistently harbor DENV and serve as replication machinery for the virus. Neverthless, other cells were found to show a varying degree of sensitivity to DENV infection and were not detected to be DENV positive in all autopsy studies. Also, few other organ-specific cells were found to be DENV positive, showing a strong potential to enable DENV replication (Figure 2). Hence further studies would exaggerate our insight into the tropism of DENV in regard to these cells and help in identifying its new replication source/s.

There are few limitations of autopsy-based DENV tropism studies which need to be highlighted: (1) The studies performed on DENV-infected cadaver indicate the organs and their respective cells that DENV infects in atypical conditions, when DENV overcomes the immune system and leads to DHF or DSS. However, in the majority of the cases, DENV infection is self-healing and is cleared from the body. Therefore, the autopsy studies do not indicate the tropism of DENV in major cases of DENV infection. (2) The contradictory results obtained during the tropism studies of various organs (brain, thymus, heart, kidney, GI tract and bone marrow) can be explained depending on the fact that the severity of DENV infection depends on multiple factors such as virus strain and its virulence, age of the individual, sex, high BMI, immune status and host genetics [1,5,6,7]. Similarly, the above factors along with the sample preparation technique, and the stage (primary or secondary) and severity (DF, DHF or DSS) of the infection when the patient died may also contribute to DENV tropism. (3) The autopsy studies do not enrich our knowledge regarding the infectious stage/severity-specific tropism of DENV which is important to understand in order to arrest DENV from further proliferation. The majority of the deaths due to DENV infection occur because of the inability to detect the extent of the infection. Hence, identifying the key markers of the different stages of DENV infection at the molecular level or at the cellular/tropism level is needed to arrest DENV infection. Further studies on alternative infection models such as humanized mouse models might help to identify the exact target cells of DENV in various organs depending on the stage or severity of the infection [118].

## Figures and Tables

**Table 1:** Overview of DENV-infected cells in humans as identified in human autopsy and various in vitro studies.

Human Organs/Tissues	Primary Cells/Cell Lines	Presence of DENV Antigens/RNA	References
**Skin**	a. Primary epidermal keratinocytes and HaCaT cells	Negative sense (−) RNA, envelope (E) and non-structural (NS) proteins NS5 and NS3	[28,121,122]
	b. Primary dermal mast cells, HMC1 and KU812 cell lines	Positive-sense (+) RNA and viral capsid (C)	[29,30,31,123]
	c. Dermal DCs (CD1c+ and CD14+) and Langerhan cells	NS1, E and positive-sense (+) RNA	[21,25,27]
	d. Monocyte-derived DCs in dermis	NS1 and E	[25,26,124]
	e. Dermal macrophages	Positive-sense (+) RNA and E	[21,27,125,126,127]
	f. Fibroblasts	NS3	[28]
	g. Lymphatic endothelium	NS3	[28]
	h. Human microvascular endothelium line-1 (HMEC-1)	E and NS1	[128,129,130,131,132,133]
**Draining Lymph nodes**	a. Macrophages	DENV RNA and NS3	[13,18,34]
	b. Immunoblasts	Unspecified	[34]
	c. Lymphocytes	Unspecified	[34]
	d. Plasma cells	Unspecified	[34]
**Spleen**	a. Macrophages in red pulp	Negative-sense (−) and positive-sense (+) RNA, NS3, E and NS1	[13,14,15,18,20,34,35,37]
	b. Splenic endothelium and sinusoidal endothelium	NS3	[18,130,131]
	c. Immunoblasts in white pulp	Positive-sense (+) RNA and an unspecified DENV antigen	[15,34]
	d. Lymphocytes in white pulp	Positive-sense (+) RNA and an unspecified DENV antigen	[15,34]
	e. Lymphocytes in red pulp	Positive-sense (+) RNA and an unspecified DENV antigen	[15]
	f. Giant cells (binucleated or multinucleated)	Positive-sense (+) RNA and an unspecified DENV antigen	[15]
	g. Plasma cells	Unspecified	[34]
	h. Mononuclear cells (macrophages and DCs) in white pulp	NS3, NS1 and E	[18,35]
	i. Germinal centers in lymphoid follicle	Positive-sense (+) RNA and an unspecified DENV antigen	[15]
	j. Centroblasts in white pulp	Positive-sense (+) RNA and an unspecified DENV antigen	[15]
**Central Nervous System**	a. Vascular endothelium and human brain microvascular endothelial cells (HBMEC)	Positive-sense (+) RNA	[74,130,131,133,134]
	b. Neurons	Positive-sense (+) RNA and NS3	[34,74,133]
	c. Astrocytes	Positive-sense (+) RNA and NS3	[18,34,74,135]
	d. Microglia	Positive-sense (+) RNA	[34,74]
	e. Lymphocytes, Purkinje cells and granular cells	Unspecified	[34]
**Kidney**	a. Immunoblasts	Unspecified	[34]
	b. Histiocytes	Unspecified	[34]
	c. Plasma cells	Unspecified	[34]
	d. Lymphocytes	Unspecified	[34]
**Liver**	a. Hepatocytes, HepG2, Huh7, Huh75.1 and Huh7.5	Negative-sense (−) and positive-sense (+) RNA and NS3	[13,14,20,34,135,136,137,138,139]
	b. Kuppfer cells	Negative-sense (−) and positive-sense (+) RNA and NS3	[13,14,15,20,34]
	c. Vascular/sinusoidal endothelium	Negative-sense (−) and positive-sense (+) RNA and NS3	[13,14,15,20,34,130]
**Lung**	a. Alveolar macrophages	Negative-sense (−) RNA and NS3	[14,15,20,34]
	b. Type II pneumocytes	Negative-sense (−) RNA and NS3	[14,20,34]
	c. Vascular endothelium and human pulmonary endothelial cell (EC) line (HPMEC-ST1.6R)	Negative-sense (−) and positive-sense (+) RNA and NS3	[14,15,20,34,130,140]
**Intestine**	a. Plasma cells in lamina propria	Unspecified	[34]
	b. Mononuclear cells in lamina propria	Unspecified	[34]
**Heart**	a. Myocardial endothelium	Negative-sense (−) RNA and NS3	[20,104]
	b. Myocardial interstitial cells	Unspecified	[104]
	c. Cardiomyocytes	Negative-sense (−) RNA and NS3	[20,104]
	d. Monocytes/macrophages	Negative-sense (−) RNA and NS3	[20]
**Stomach**	a. Lymphocytes in mucosa	Unspecified	[34]
	b. Plasma cells in lamina propria	Unspecified	[34]
**Blood**	a. Peripheral blood monocyte/macrophage (PBMC)-derived primary B cells and B cell lines (Raji cells, Wil 2WT, BM, LK63, Daudi and 8866)	Negative-sense (−) RNA, an unspecified DENV antigen (primary B cells) and DENV RNA (BM and LK63, Daudi, Raji)	[141,142,143,144,145]
	b. PBMC-derived T cells and T cell lines (JK44, JK49, CB2.8, CB6.17, HSB-2, Molt-4 and Jurkat)	(Wil 2WT and 8866) DENV NS3, C, NS1 (Primary T cells), DENV RNA (Molt-4 and Jurkat) and an unspecified antigen (JK44, JK49, CB2.8, CB6.17 and HSB-2)	[143,145,146,147]
	c. Activated monocytes and U937 cells	prM and NS3	[17,125,127,128,148,149,150]
	d. Blood-derived DCs	Negative-sense (−) and positive-sense (+) RNA, DENV E and NS1	[25,124,126,151,152]
